# Prevalence and associated factors of visual impairment among adults aged 40 and above in Southern Ethiopia, 2022

**DOI:** 10.1038/s41598-024-53056-6

**Published:** 2024-01-30

**Authors:** Tamiru Getachew Deme, Masresha Mengistu, Firdawek Getahun

**Affiliations:** 1 School of Medicine, Department of Human Anatomy, Arba Minch University, Arba Minch, Ethiopia; 2 Department of Ophthalmology, Arba Minch General Hospital, Arba Minch, Ethiopia; 3 School of Public Health, Arba Minch University, Arba Minch, Ethiopia

**Keywords:** Diseases, Eye diseases

## Abstract

Visual impairment is a functional limitation of the eye caused by a disorder or disease that can reduce one’s ability to perform daily activities. Many studies in Ethiopia have focused on childhood visual impairments. We assessed the prevalence and factors associated with visual impairment among adults aged 40 and above. Community-based cross-sectional study was done and a systematic sampling technique was used to select 655 participants. Data were collected by interviewer administered questionnaire, E-Snell chart, pinhole, torch light, and magnifying loupe. SPSS version 25 was used for analysis. Bivariate and multivariate analyses were performed to identify factors associated with outcome variable. The overall prevalence of visual impairment was found to be 36.95% (95% CI 33.2–40.8%). Factors associated with a higher odds of visual impairment included aged 51–60 years (AOR 2.37, 95% CI 1.29–4.44), aged 61 and above (AOR 8.9, 95% CI 4.86–16.3), low wealth index (AOR 1.91, 95% CI 1.14–3.2), divorced and widowed (AOR 4.67, 95% CI 2.77–7.86), no formal education (AOR 14.28, 95% CI 2.82–71.46), not utilizing eyeglass (AOR 3.94, 95% CI 1.65–9.40). The prevalence of visual impairment was relatively high compared to other studies. Age, marital status, occupation, educational status, wealth index, and not using eyeglasses were significantly associated with visual impairment. Refractory error is the leading cause of visual impairment. Early eye care service interventions are needed in this area.

## Introduction

Visual impairment (VI) is a functional limitation of the eye or visual system due to a disorder or disease that can reduce one’s ability to perform activities of daily living^1^. According to the World Health Organization (WHO) revised definition, VI refers to a presenting distance visual acuity (PVA) that is worse than 6/18 in worst eye^2^. The Snellen ‘E’ chart is commonly utilized to assess visual acuity at a distance of 6 m^3^.

Visual impairment causes disabilities by significantly interfering with one’s ability to function independently. These disabilities limit personal and socioeconomic independence, and a visual handicap exists^4^. Visually impaired elderly individuals are at increased risk of falls, fractures, and depression^5^. Hence, their ability to find employment, support themselves, and provide for their families is diminished^6^. More than two-thirds of visual impairment and blindness can be avoided by either prevention or treatment^7^.

The burden of VI is not distributed uniformly throughout the world, with the least developed regions having the largest share. It is also unequally distributed across age groups^8^. Most people with vision impairment and blindness are over the age of 50; however, vision loss can affect people of all ages^7^. Adults age 40 and above are at risk of developing serious eye diseases leading to vision loss^9^. As populations continue to age, the prevalence of vision impairment and blindness are projected to more than double for the next 30 years^10^.

Previous studies have shown that several factors are associated with visual impairment, such as older age^11^, rural residency^12^, lower educational status, low monthly income^12^, cataracts^11,13,14^, glaucoma^13^, macular degeneration^13^, chronic comorbid illnesses^11^ and smoking^15^.

According to a global WHO report, about 2.2 billion people have distance VI. Almost half of these cases have been prevented or are yet to be addressed. The leading causes of VI and blindness are found to be uncorrected refractive error and cataracts^7^. The prevalence of VI among adults aged 40 years in the South Indian, State of Andhra Pradesh was 14.3%^16^ and in East Delhi district on similar age category was 11.4%^17^. The data on the prevalence of visual impairment in Africa varies. In Ghana among Farmers aged ≥ 40 years was 22.7%^18^. In Upper Egypt among adults aged ≥ 40 years was 38.8%^19^.

In Ethiopia many studies were conducted previously on similar topics, however, most of them focused on childhood visual impairment^20,21^. Besides, the studies included participants aged cut of point at 18 years and above. A study conducted in Debre Markos, Ethiopia the magnitude of VI was 36.52%^22^, at St Paul’s Millennium Hospital Medical College, Ethiopia, low Vision and Blindness was found to be 10.3% and 7.3%, respectively^23^ and a community based study using worst eye seeing visual acuity in Debre Berehan town, Ethiopia among aged 18 and above the prevalence of visual impairment was found to be 16.8%^24^.

The high incidence of eye disease in Ethiopia is believed that it has brought significant economic and social consequences for individuals, society, and the nation^25^. There are also impacts on caregivers, such as children who can't go to school and adults who are out of work^26^. Although multiple approaches and strategies to decrease the incidence of visual impairment conducted in Ethiopia by promoting the use of eye glasses, increasing public awareness, and free mass campaign service for cataract and trichiasis surgery, blinding factors are still on the rise due to the growing population and aging^27^.

Most visual impairments are caused by uncorrected refractive errors, which can be easily corrected by wearing eye glasses^13^. Uncorrected visual problems may affect education, employment opportunities, productivity, and quality of life. The early detection and treatment of visual problems will reduce the dependency and burden of the disease on society. Understanding the prevalence and associated factors will help control and prevent visual impairment. To plan health services or for risk factor analysis, it is necessary to know the prevalence and distribution of visual impairment in community-dwelling populations.

To the best of our knowledge, no previous study has been conducted in Ethiopia in general or in a study area, in particular, by including adults aged 40 and above who are at high risk for developing visual problems.

## Methods and materials

### Study settings, design, and population

A community-based cross-sectional study was conducted among residents living in the Arba Minch Zuria District in Southern Ethiopia from October to November 2022 in nine kebeles (the lowest administrative unit of Ethiopia). The district is bordered to the South by the Dirashe Special District, to the West by Bonke District, to the North by Dita and Chencha Districts, to the Northeast by Mirab Abaya District, to the East by the Oromia Regional State, and to the Southeast by the Amaro Special District. The Arba Minch Zuria district has 31 kebeles with three different climatic zones: highlands, midlands, and lowlands. According to Ethiopian statistical service data, the total projected population size of the area for the year 2023 is 217,560, among which male accounts 108,691 and female, 108,869^28^. According to the woreda health office, ten health centers and 33 health posts provide health services for the community.

*Source population* All adults aged 40 and above who lived in Arba Minch Zuria district.

*Study population* All adults whose age 40 years or above who fulfilled the inclusion criteria. Respondents who were unable to speak, were severely ill, or had recent ocular trauma or surgery were excluded.

### Sample size determination and sampling procedure

The sample size was calculated using the single population proportion formula for cross-sectional study by considering the following assumptions: P (Prevalence of VI among adults at Debre Berhan town was 16.8%^24^ α (level of significance)** = **5%, The Z value at 95% CI and 5% α = ± 1.96 (two tailed), Margin of error (W) = 0.03 and “n” is the required sample size.

$$\begin{aligned} {\text{n}} & = \frac{{{\text{Z}}(1 - \upalpha /2)^{2} {\text{p}}\left( {1 - {\text{p}}} \right)}}{{{\text{W}}^{2} }} \\ {\text{n}} & = \frac{{\left( {1.96} \right)^{2} *0.168*0.832}}{{\left( {0.03} \right)^{2} }} = 596 \\ \end{aligned}$$

Then, by adding a 10% non-response rate, the final sample size was 655.

Nine kebeles in the Arba Minch Zuria Woreda were randomly selected. The total study population Households (HHs) with adults aged ≥ 40 and above were obtained from each kebeles. The total sample size was distributed to each of the selected kebeles using proportional allocation to sample size. Households were systematically selected depending on the total number of households required for each kebele by dividing the number of households in each kebele by the sample size. One participant was randomly selected from each household. However, if there were no eligible subjects in the selected household, the next immediate neighbor’s household with eligible study subjects was included.

### Study variables

*Dependent variable* Visual impairment.

*Independent variables* were sex, age, level of education, occupation, marital status, residence, and other relevant information related to visual impairment, such as eyeglasses, flashlight exposure, cigarette smoking, alcohol use, previous ophthalmic clinic visit, chronic comorbidity, history of eye trauma, family history of eye disease, and history of eye disease.

### Operational definitions

*Presenting visual acuity* defined as a distance visual acuity without any correction in each eye^2^.

*Normal vision* a presenting visual acuity > 6/18 in the better eye^2^.

*VI* was considered for this study when PVA was less than 6/18 in the worst eye^24^.

*Bilateral VI* defined as visual acuity of < 6/18 in the better eye^29^. It included:

*Bilateral moderate VI*, defined as visual acuity of < 6/18 and > 6/60 in the better eye^29^;

*Bilateral severe VI*, defined as a visual acuity < 6/60 and > 3/60 in the better eye^29^; and *Bilateral blindness*, defined as a visual acuity < 3/60 and NLP in the better eye^29^.

*Unilateral VI* was defined as visual acuity worse than 6/18 in one eye but better than or equal to 6/18 in the other eye^30^.

*Monocular moderate VI* was defined as PVA < 6/18 to ≤ 6/60 in one eye and 6/6 to ≤ 6/18 in the other eye^31^.

*Monocular severe VI* was defined as PVA < 6/60 to ≤ 3/60 in one eye and 6/6 to 6/60 in the other eye^31^.

*Monocular blindness* was defined as PVA < 3/60 to NLP in one eye and PVA 6/6 to 3/60 in the other eye^31^.

*Uncorrected refractive error* when the presenting visual acuity was less than 6/18, but improved to 6/18 or better with pinhole vision^31^.

*Cataract* Opacity of the crystalline lens in the pupillary area as observed with torchlight and loup.

*Trachoma* marked in cases with central corneal scarring in the presence of at least one of the following signs of trachoma: trichiasis/entropion ^32^.

*Other causes of VI* include all causes other than those mentioned above.

*Eye trauma* Self-reported previous history of any trauma to the eye.

*Eye checkup* If the participants visited the health facility at least once in the past 2 years for eye examination^33^.

*Family history of eye disease* Participants with a positive history of vision problems in their family members/near relatives (parents and grandparents).

*Flashlight exposure* Occupational exposure to radiation reflected from metal welding^34,35^.

*Substance use* Use of at least one substance (alcohol or cigarettes) in an individual’s lifetime^36^.

*Current user* A person who consumed any substance at least once within the last 30 days^36^.

*Ever use* Use of any substance at least once in an individual’s lifetime^36^.

*Wealth index* it is a composite indicator for measuring the living standard of households^37^.

### Data collection procedure and collection instrument

Data were collected using an interviewer-administered structured questionnaire and observational checklist developed in different studies. The questionnaire contained the following items: socidemographic, behavioral, and environmental characteristics and previous medical history and comorbidities. Specifically, the wealth index assessing questionnaire included: household’s ownership of a selected set of assets, housing characteristics, type of water access, and toilet and sanitation facilities. The checklist contained an assessment of VI and clinical characteristics.

Data were collected using the Kobo toolbox. Clinical examination was conducted using Snellen’s “E” optotype chart, pinhole, torch light, and a 2.5× magnifying loupe. The data collection teams included three optometrists, nine diploma-holding nurses, ophthalmologists, and four MSc/MPH holder supervisors. After obtaining written informed consent from the study participants, optometrists measured the PVA using Snellen’s “E” optotype chart at 6 m for each eye, separately. This measurement has 0.73 sensitivity and a specificity of 0.93 in previous studies^38^. The procedure was conducted outdoors in the shade on both bright and sunny days. Visual acuity < 6/18 in the worst eye was considered VI. Adults with PVA of less than 6/18 in the worst eye underwent comprehensive eye examination by optometrists to determine the possible causes of VI.

Using a torch light and magnifying loupe, each eye was tested separately for in-turned lashes (trichiasis), the cornea was inspected for corneal opacities, and the lens was examined for cataracts. An individual with PVA < 6/18 and an improvement of PVA with pinholes was confirmed as VI due to refractive error. If a person wore spectacles, the pinhole was placed in front of them. In some cases, the available correction was not optimal. Vision with pinhole correction cannot be worse than presenting vision.

The visually impaired participants who had undetermined eye problems were referred to an ophthalmologist for a detailed eye examination. The causes of VI were recorded for each eye separately. In a possible scenario of two causes of VI presented for each eye, the one that could be more avoidable, that is either preventable or treatable, was chosen^29^. All participants with VI were linked to the Arba Minch General Hospital Ophthalmology Center for appropriate management and follow-up. Specifically, participants who developed cataracts were treated at Arba Minch General Hospital through mass-campaign-free cataract surgery.

Ethical approval was obtained from the Arba Minch University Institutional Ethical Review Board (IRB/1221/2021). Written consent was obtained from all the selected households and individual participants. Individuals with VI were referred to an ophthalmologist at the Arba Minch General Hospital for detailed eye examination. Moreover, this study was conducted in accordance with the Declaration of Helsinki, and all ethical and professional considerations were maintained throughout the study to keep participants’ data strictly confidential.

### Data processing and analysis

After checking the completeness and consistency of the data, it was entered into Excel and exported to SPSS version 25 for analysis. Descriptive statistics, frequency distributions, and percentages were calculated for categorical data and are shown by using pie charts, bar graphs, and tables. Principal component analysis was performed to generate a wealth index.

Binary logistic regression was performed to identify candidate variables associated with visual impairment. In a multi-variant analysis, the variables with *p* < 0.25 in the bivariant analysis, were included and adjusted OR with 95% CI was computed. Variables with *p* < 0.05 were considered significantly associated with visual impairment. The variance inflation factor (VIF) and tolerance test were checked for multicollinearity, with values ≥ 0.1 and < 10, respectively, to control for confounders. Then, the Hosmer–Lemeshow goodness-of-fit test was performed to check for model fitness.

### Data quality assurance

Data cleaning was performed to assess completeness, consistency, outliers, and missing values. Two days of training were provided to data collectors and supervisors on data collection tools, the purpose of the study, data collection skills, and ethical procedures. Pretests were performed on a 5% sample size from outside the study area, and necessary corrections were made. By taking 5% of the collected data randomly, the consistency was cross-checked. Any errors identified during the review were corrected accordingly by supervisors and investigators. During the data collection period, 5% of the data were cross-checked daily for completeness by the principal investigator. Interobserver agreement among optometrists for distant visual acuity testing was determined, and Cohen’s kappa was found to be 0.95.

### Ethical approval and consent to participate

Ethical approval was obtained from the Arba Minch University Institutional Ethical Review Board (IRB/1221/2021). A letter of permission to undertake the study was secured from the Gamo Zone Health Department, respective woreda, and kebele. Written consent was obtained from all the selected households and individual participants. Individuals with VI were referred to an ophthalmologist at the Arba Minch General Hospital for detailed eye examination. Individuals who had confirmed cases of cataracts were treated at the Arba Minch General Hospital during a free cataract surgery campaign held in February 2022. This study was conducted in accordance with the Declaration of Helsinki. COVID-19 preventive measures were ensured during the data collection. Confidentiality of participants’ information was secured.

## Results

### Socio-demographic characteristics of the participants

Of the respondents, 655 participated in the study, making a response rate of 100%. Of the participants, 384 (58.63%) were male. The mean age of the participants was 58.81 ± 0.34 (SD) and more than two fifths (276; 42.114%) of them were in the age group between 60 and 69 years. The majority of them (555; 84.73% and 523; 79.85%) were married and had no formal education, respectively. Also, 417 (63.6%) of the participants were farmers. One-fifth of the respondents had the lowest and highest wealth indices (Table 1).

**Table 1:** Socidemographic characteristics of the study participants (n = 655) in Southern Ethiopia.

Variable	Categories	Frequency	Percent
Age category	40–49 years	101	15.42
	50–59 years	201	30.69
	60–69 years	276	42.14
	70 and above	77	11.76
Gender	Male	384	58.63
	Female	271	41.37
Marital status	Married	555	84.73
	Divorced	8	1.22
	Widowed	92	14.05
Educational status	Able to read and write	48	7.33
	Grade 1–8	60	9.16
	Secondary and above	24	3.66
	No formal education	523	79.85
Religion	Orthodox	196	29.92
	Protestant	445	67.94
	Muslim	2	0.31
	Other	13	1.98
Occupation	Farmer	417	63.66
	House wife	166	25.34
	Goverment and retired	39	5.95
	Others	33	5.04
Ethnicity	Gamo	573	87.48
	Wolayta	24	3.66
	Amhara	52	7.94
	Others	6	0.92
Wealth index	Lowest	131	20
	Second	131	20
	Middle	131	20
	Fourth	131	20
	Highest	131	20

### Previous medical history and comorbidities

One eighth (82, 12.52%) of the participants had a history of eye problems. Among those with a history of eye problem, 25 (30.48%) had a known history of cataracts. Twenty-five (3.82%) of the participants had a known history of hypertension (Table 2).

**Table 2:** Previous medical history and comorbidities of the study participants in Southern Ethiopia.

Variable	Categories	Frequency	Percent
Family history of eye disease	Yes	19	2.9
	No	636	97.1
History of eye problem	Yes	82	12.52
	No	573	87.48
Known history of cataract	Yes	25	30.48
	No	57	69.51
Known history of diabetes	Yes	10	1.53
	No	645	98.47
Known history of hypertension	Yes	25	3.82
	No	630	96.18

### Prevalence of visual impairment, clinical characteristics and possible causes

The overall prevalence of visual impairment was found to be 36.95% (95% CI 33.2–40.8%). Among the overall visual impairment participants, 151 (62.4%) and 91 (37.6%) had unilateral and bilateral visual impairment, respectively. Thirty-three (36.26%) and 60 (39.74%) had bilateral and unilateral severe visual impairments, respectively (Table 3).

**Table 3:** Prevalence and clinical characteristics of visual impairment of the study participants in Southern Ethiopia.

Variable	Categories	Frequency	Percent
Presence of visual impairment (n = 655)	Yes	242	36.95
	No	413	63.66
Category of visual impairment	Unilateral (PVA < 6/18 in worst eye)	151	62.40
	Bilateral (PVA < 6/18 in better eye)	91	37.60
Severity of bilateral visual impairment (n = 91)	Moderate VI	50	54.95
	Sever visual impairment	33	36.26
	Blindness	8	8.79
Severity of unilateral visual impairment (n = 151)	Moderate VI	84	55.63
	Sever visual impairment	60	39.74
	Blindness	7	4.64

**Figure 1:**
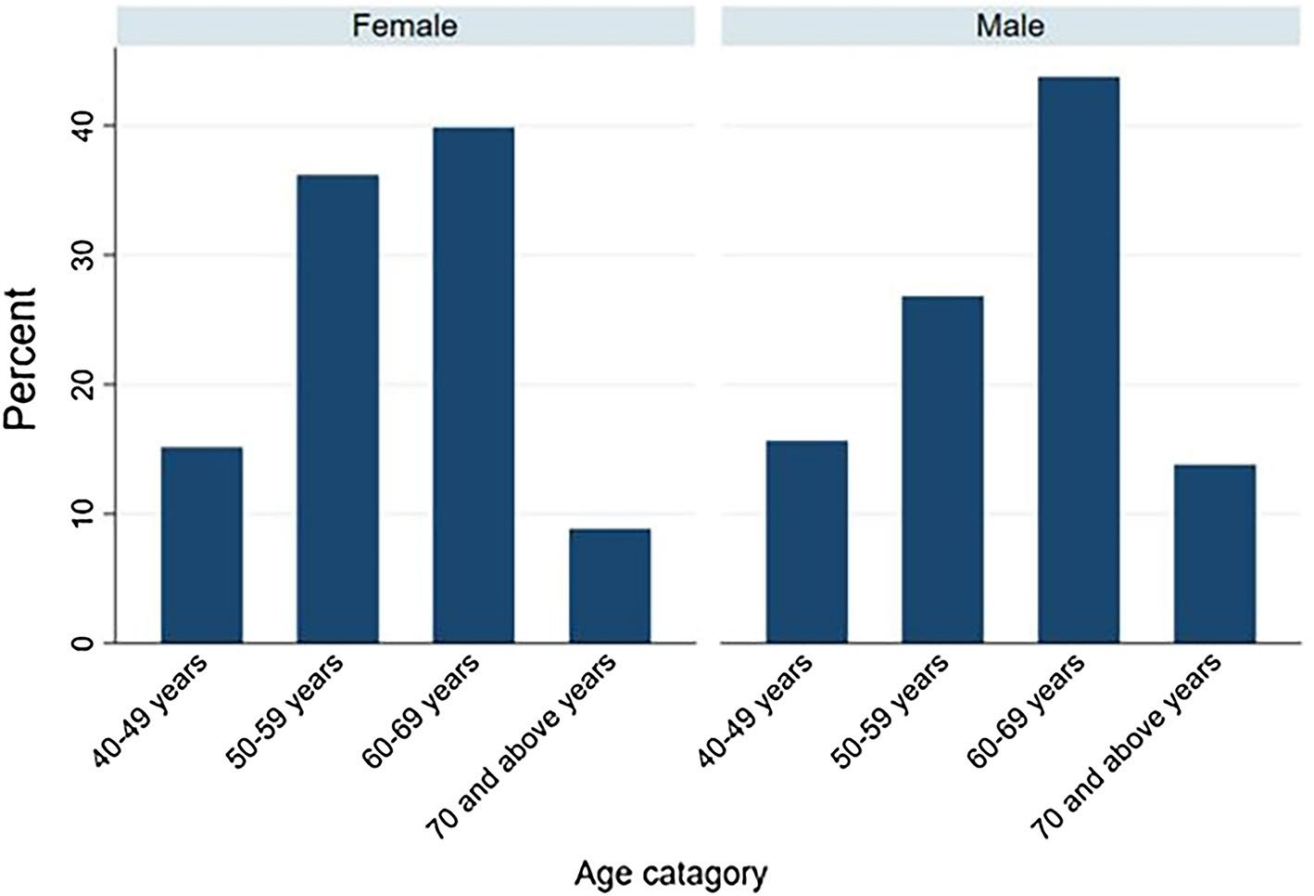
Prevalence of visual imapriment by sex and age category of the study participants in Southern Ethiopia.

Among females who had VI, more than one third (47/120, 38.5%) were in the age group between 60 and 69 years. Similary, 54/122 (44.26%) of the males developed VI in a similar age category. Only 15% (18/120) and 15.57% (19/122) of females and males developed VI in the 40–49 age group, respectively (Fig. 1).

**Figure 2:**
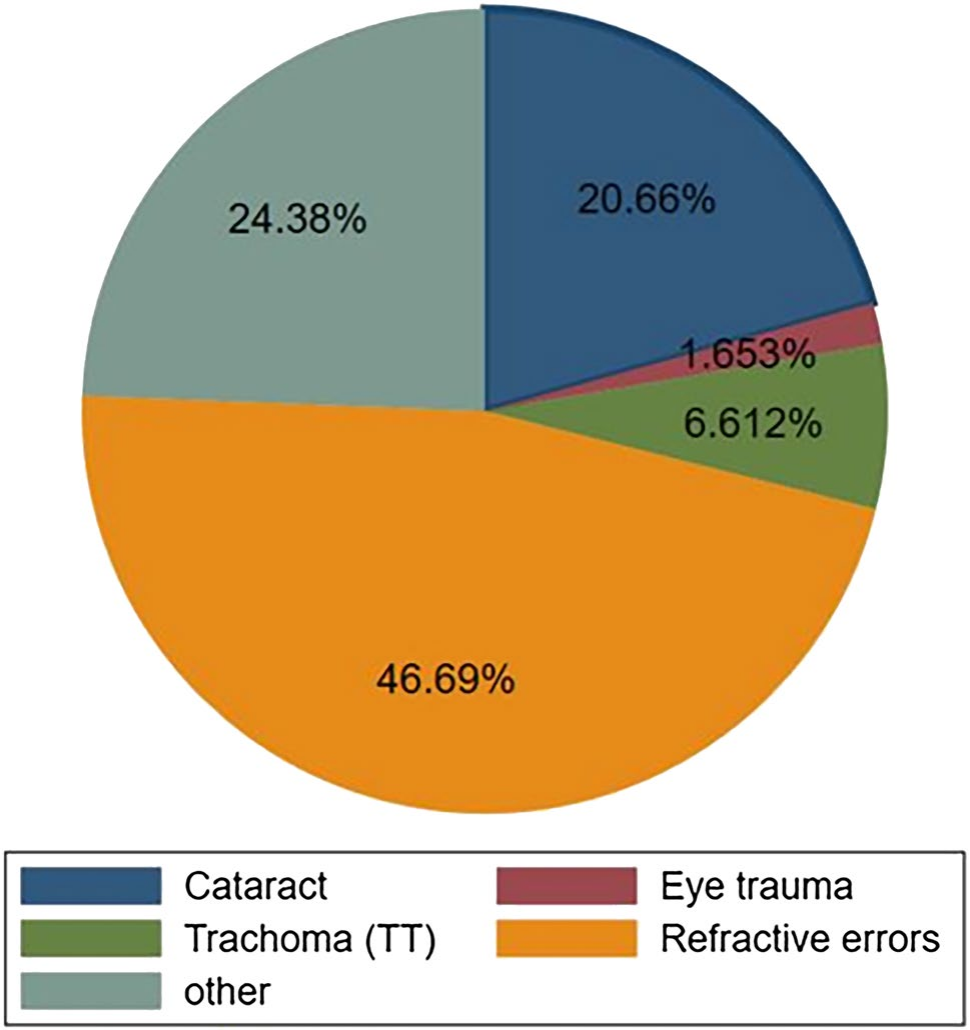
Possible cause of visual impairment among adults aged 40 and above in Southern Ethiopia.

The most common possible cause of visual impairment in this study was found to be refractive error (113; 46.69%), followed by unknown causes (59; 24.38%) and cataract (50; 20.6%) (Fig. 2).

### Behavioral and environmental characteristics

The results showed that 131 (20%) of the participants had used a substance during their lifetime. Twenty-seven (4.12%) of the participants used the prescribed eyeglasses. More than half of the study participants (14; 51.85%) utilized distant type eye glasses. About two-thirds (18; 66.67%) of the respondents had worn eye glasses for 1–5 years Regarding the frequency of wearing eyeglasses, more than three quarters (21; 77.78%) of the participants wore eye glasses sometimes. Almost all (646; 98.63%) of the participants had no regular history of eye checkups. More than half (332; 50.69%) of the respondents had to walk more than 30 min to get water. In total, 588 (89.77%) of the participants were practicing throw-out method of garbage disposal. (Table 4).

**Table 4:** Behavioral and environmental characteristics of the study participants in Southern Ethiopia.

Variable	Categories	Frequency	Percent
Have you ever used any substance (alcohol, cigarate) in your life time?	Yes	131	20
	No	524	80
Alcohol consumption	Current drinker	48	7.3
	Ever drinker	54	8.2
	Never drinker	553	84.4
Smoking cigarette	Current smoker	14	2.1
	Ever smoker	29	4.4
	Never smoker	612	93.4
Exposure to flight-light	Yes	2	0.31
	No	653	99.69
Wearing eye glass	Yes	27	4.12
	No	628	95.88
Type of eye glass	Distant	6	22.22
	Photochromic	7	25.93
	Reading	14	51.85
Source of eye glass service	Government hospital	10	37.04
	NGO	3	11.11
	Private eye specialists	4	14.81
	Illegals’ shop	10	37.04
Duration of eyeglass utilization	1–5 years	18	66.67
	5–10 years	3	11.11
	Less than 1 year	5	18.52
	More than 10 years	1	3.70
How often do you wear eye glass?	Always	4	14.81
	Sometimes	21	77.78
	Usually	2	7.41
History of eye check up	Yes	9	1.37
	No	646	98.63
Source of water source	Pipe	434	66.26
	River/stream	214	32.67
	Borehole/well	7	1.07
Average distance from water source	Far away from home (more than 30 min)	332	50.69
	Near home (less than 30 min)	323	49.31
Type of toilet facility	Pit latrine without slab/open pit	634	96.79
	No facility/bush field	12	1.83
	Pit latrine with slab	9	1.37
Method of garbage disposal	Burn	67	10.23
	Throw out	588	89.77
Types of fuel used for cooking	Cubes	169	25.8
	Wood	477	72.82
	Electricity	9	1.37

### Factors associated with visual impairment

Sex, age category, marital status, occupation, educational status, wealth index, substance use, a known history of hypertension, wearing eyeglasses, having regular eye checkups, and sources of water were found to be associated with visual impairment in bivariate analysis at *p* < 0.25.

In the multivariate logistic regression analysis, age, marital status, occupation, educational status, wealth index, and wearing eyeglasses were significantly associated with visual impairment.

The odds of visual impairment were three times higher in adults aged 51–60 years and nine times higher in adults aged 61+ years (AOR 2.37, 95% CI 1.29–4.33; AOR 8.9, 95% CI 4.86–16.3), respectively, compared to adults aged 40–50 years. Those participants who were Farmers were 43% less likely to develop visual impairment than those who were government employees (AOR 0.57, 95% CI 0.38–0.86). The development of visual impairment among participants in lowest wealth index was more likely than those in the highest wealth index (AOR 1.91, 95% CI 1.14–3.2).

Participants who were divorced or widowed were nearly five times more likely to develop visual impairment than those who were married (AOR 4.67, 95% CI 2.77–7.86). The odds of developing visual impairment among respondents who had no formal education, were able to read and write, and had completed grades 1–8 were AOR 14.28 (95% CI 2.82–71.46), AOR 8.15 (95% CI 1.4–46.63), and AOR 6.95, 95% CI 0.287–37.6), compared to those who had secondary and above educational status, respectively. Visual impairment among participants who did not wear the prescribed eye glasses were four times more likely to have visual impairment than their counterparts (AOR 3.94, 95% CI 1.65–9.40) (Table 5).

**Table 5:** Bivariate and multivariate logistic regression for factors associated with visual impairment among adults aged 40 and above in Southern Ethiopia.

Variable	Category	Visual impairment		COR (95%CI)	AOR (95%CI)	*p* value
		Yes	No			
Sex	Female	120 (49.59%)	151 (36.56%)	1.71 (1.23–2.35)	1.341 (0.56–3.18)	0.72
	Male	122 (50.41%)	262 (63.4%)	1	1	
Age category	40-50 years	17 (7.02%)	121 (29.30%)	1	1	
	51–60 years	71 (29.34%)	191 (46.28%)	2.64 (1.48–4.707)	2.31 (1.29–4.33)	0.007*
	61 and above years	154 (63.64%)	101 (24.46%)	10.85 (6.16–19.11)	8.9 (4.86–16.37)	0.000*
Marital status	Married	173 (71.49%)	382 (92.49)	1	1	0.000*
	Widowed and divorced	69 (28.51%)	31 (7.51%)	4.91 (3.10–7.78)	4.67 (2.77–7.86)	
Occupation	Farmer	152 (62.81%)	265 (64.16%)	0.66 (0.34–1.29)	0.57 (0.38–0.86)	0.008*
	House wife	57 (23.55%)	109 (26.39%)	0.61 (0.30–1.236)	0.6 (0.15–2.36)	0.46
	Government employee	18 (7.44%)	21 (5.08%)	1	1	
	Others**	15 (6.2%)	18 (4.36%)	0.97 (0.38–2.46)	1.42 (0.402–5.025)	0.585
Educational status	No formal education	213 (88.02%)	310 (75.06%)	3.43 (1.157–10.19)	14.28 (2.82–71.46)	0.001*
	Able to read and write	11 (4.55%)	37 (8.96%)	1.48 (0.419–5.28)	8.15 (1.4–46.63)	0.018*
	Grade 1–8	14 (5.79%)	46 (11.14%)	1.52 (0.445–5.201)	6.95 (1.287–37.55)	0.024*
	Secondary and above	4 (1.65%)	20 (4.84%)			1
Wealth index	Lowest	58 (23.97%)	73 (17.68%)	1.24 (0.76–2.03)	1.91 (1.14–3.2)	0.014*
	Second	47 (19.42%)	84 (20.34%)	0.87 (0.53–1.44)	0.83 (0.467–1.48)	0.534
	Middle	45 (18.6%)	86 (20.82%)	0.82 (0.496–1.35)	0.71 (0.39–1.29)	0.261
	Fourth	41 (16.94%)	90 (21.79%)	071 (0.42–1.18)	0.69 (0.38–1.25)	0.227
	Highest	51 (21.07%)	80 (19.37%)	1		
Substance abuse	Yes	58 (23.97%)	73 (17.68%)	1.46 (0.99–2.16)	1.71 (1.03–2.81)	0.37
	No	184 (76.03%)	340 (82.32%)	1	1	
Known history of hypertension	Yes	14 (5.79%)	11 (2.66%)	2.24 (1.00–5.02)	2.33 (0.82–6.61)	0.11
	No	228 (94.21%)	402 (97.34%)	1	1	
Having regular eye check up	Yes	6 (2.48%)	3 (0.73%)		1	
	No	236 (97.52%)	410 (99.27%)	3.485043 (1.680–7.228	0.17 (0.027–1.05)	0.057
Wearing prescribed eye glass	Yes	9 (3.72%)	49 (11.86%)	1		
	No	233 (96.28%)	364 (88, 14%)	3.48 (1.68–7.23)	3.94 ((1.65–9.40)	0.002*
Source of water	Borehole/well	2 (0.83%)	5 (1.21%)	0.48 (0.107–2.14)	1.45 (0.23–9.08)	0.68
	Pipe	172 (71.07%)	262 (63.44%)	1	1	
	River/stream	68 (28.10%)	146 (35.35%)	1	1.03 (0.66–1.61)	0.88

*Significant at P<0.05.

## Discussion

This study was conducted to assess the prevalence and factors associated with VI among adults aged ≥ 40 years. To the best of our knowledge, this is the first study to assess VI among adults aged ≥ 40 years in Ethiopia.

The prevalence of visual impairment among adults aged ≥ 40 years was found to be 36.95% (95% CI 33.2–40.8%). Among visually impaired participants, 151 (62.40%) and 91 (37.60%) had monocular and bilateral visual impairment, respectively.

The prevalence of visual impairment was higher than in studies conducted in Saudi Arabia (13.9%)^39^, South Korea (4.1%)^40^, India (8.4%)^41^ South Sudan (11.8%)^42^, South Africa (4.9%)^43^ Debre Berehan (16.8%)^24^ and Gondar, Ethiopia (15.3%)^44^. This discrepancy might be due to age differences: in the Saudi Arabia and Debre Berehan studies: the participants were aged 18 years and above. As shown in this study, age was significantly associated with visual impairment. This difference could also be due to the study setting, for instance, the South Africa, Debre Berehan, and Debre Markos studies were conducted in urban areas, but the current study was conducted in rural areas. Rural dwellers are more likely to be exposed to visual impairment, which could be due to a lack of awareness of health-related issues and poor healthcare accessibility^22^.

In addition, the discrepancy might be due to the definition of visual impairment; for instance, the study in Debre Markos was conducted using better eye-presenting visual acuity, which means that it only considered bilateral visual impairment. This underestimates the prevalence of visual impairment compared to that in the present study, which considered the visual acuity of the worst eye. The studies conducted in Saudi Arabia and South Korea utilized best-corrected visual acuity, which underestimated the prevalence of VI as it excluded VI caused by refractor error. This discrepancy might also be due to differences in technological advancement, awareness, and healthcare facilities.

The finding of this study is lower than the overall prevalence of studies conducted in China (49.7%) and Russia (64.7%)^45^. This difference could be due to the age difference of the study participants; in Russia, study participants were aged 85 years and above, and in China they were aged 70 and above, compared to 40 and above for the current study. In addition, the cut-off point for defining VI in both studies was < 6/12. This might have overestimated the prevalence of VIs.

The prevalence of visual impairment in this study was in line with a study conducted in Debere Markos, Ethiopia (36.5%)^22^. This similarity might be due to the use of presenting visual acuity and a cut-off point of 6/18 for defining VI.

The odds of developing visual impairment among those aged 51–60 years and above 61 years were more likely to develop visual impairment than those aged 40–50 years. This finding is supported by previous studies in Saudi Arabia ^39^, South Africa^43^, China^45^, South Korea ^40^, Debere Markos^22^, Debere Berehan ^24^ and Gondar, Ethiopia (15.3%)^44^.

A possible reason for the increased visual impairment in old age might be the increase in age-related eye diseases and degeneration^3,46^. As age increases, the function of the entire body, including the visual system, becomes less efficient as a result of physiological deterioration as well as increased exposure to ocular infections due to deterioration of the eye structure, and people may suffer more eye diseases related to aging, such as macular degeneration, cataracts, and retinopathy, which leads to visual impairment^46^.

Farmers were less likely to develop visual impairment than were government employees. A possible reason for this could be that government employees are more likely to utilize computers for their work-related activities for a longer time than farmers which may cause VI. Divorced and widowed participants were nearly five times more likely to develop visual impairments. This finding is consistent with the results of a study conducted in South Korea^40^. A possible justification for this might be that divorced and widowed participants are less likely to be concerned about their health status and undergo regular health check-ups, as they might not have anyone who can consult or support them.

The odds of developing visual impairment among respondents who had no formal education, were able to read and write, and had completed grades 1–8 were more likely to develop visual impairment than those who had secondary or above educational status. Previous studies have reported similar findings^22,47,48^. This is because those who are less educated are more likely to have poor health-seeking behavior and knowledge of risk factors for VI.

In this study, we did not find VI to be associated with self-reported history of hypertension and DM. A possible explanation is that we only assessed the presence or absence of DM and hypertension but did not examine their duration or severity. A study conducted in Taiwan showed that a disease duration > 10 years for DM and hypertension was independently associated with VI^14^.

In contrast, respondents who did not wear prescribed eyeglasses were four times more likely to develop visual impairment than their counterparts. A possible explanation for this is that, as this study depicted, the leading cause of VI is refractory error, which needs to be corrected using eyeglasses. Beside, this study finding showed that approximately 42.14% of the participants were aged between 60 and 69 years which were vulnerable to acquire refractory error. The prevalence of refractory error increases with age^49^.

The odds of participants with a lower wealth index developing VI were higher than those with a higher wealth index. This finding is supported by those of previous studies^50,51^. A possible justification is that low-income participants have poor access to healthcare facilities; therefore, they are not treated early to restore their vision.

An Uncorrected Refractive Error is the leading cause of vision impairment. Despite the availability of cost-effective interventions in the form of spectacles, millions suffer from this worldwide^52^. The current study showed that refractory error was the main cause of visual impairment. This finding is in line with those of previous studies in which cataract and refractory error were the common causes of visual impairment in the aged population^53^. Another study in Saudi Arabia also showed that the main medical causes of visual impairment was refractive errors, followed by cataracts^39^. This might be due to the improper utilization of prescribed spectacles and low cataract surgery service coverage in these areas.

According to the American Academy of Ophthalmology, comprehensive eye examination is recommended every 1–2 years for adults with risk factors for VI^33^. In our study, only 1.37% of the participants had a history of eye checkups at least once in the past 2 years. In a study conducted in Hawassa, 23.8% of respondents had eye checkup examinations at least once within the past year^54^. This difference may be because our study participants were from rural areas, which may have resulted in less accessibility to health facilities. Moreover, this difference might also be due to low incomes.

### Strengths and limitations of the study

To the best of our knowledge, this study is the first in its kind in the study area and utilized optometrist nurses as data collectors to obtain high quality data. Nevertheless, the study is not without limitations. First, the diagnosis of possible causes of visual impairment was performed without ophthalmoscopy, which made us unable to assess some conditions.

Second, the definition of visual impairment was limited to distant visual acuity, whereas near distance and visual field were not assessed, which may have underestimated the prevalence of uncorrected refractive error, glaucoma, or other optic atrophies.

Third, given the cross-sectional nature of the study, our findings suggest an association between visual impairment and various factors, but not a causative relationship.

## Conclusion and recommendations

In this cross-sectional study, the prevalence of visual impairment among older adults was relatively high, and more than three-fifths of participants had unilateral visual impairment. Age, marital status, occupation, educational status, wealth index, and wearing of prescribed eyeglasses were significantly associated with visual impairment. Refractory errors are the leading cause of visual impairment. Further studies are recommended to assess the coverage of cataract surgery, spectacle utilization, and visual-related quality-of-life in individuals with visual impairment.

## Data Availability

The datasets used and/or analyzed during the current study available from the corresponding author on reasonable request.
